# "No Papers, No Treatment": a scoping review of challenges faced by undocumented immigrants in accessing emergency healthcare

**DOI:** 10.1186/s12939-024-02270-9

**Published:** 2024-09-14

**Authors:** Sezer Kisa, Adnan Kisa

**Affiliations:** 1 Department of Nursing and Health Promotion, Faculty of Health Sciences, Oslo Metropolitan University, Oslo, Norway; 2 School of Health Sciences, Kristiania University College, Oslo, Norway; 3 Department of International Health and Sustainable Development, Tulane University, New Orleans, USA

**Keywords:** Barriers, Emergency healthcare, Health equity, Policy, Public health, Undocumented immigrants

## Abstract

**Background:**

Undocumented immigrants face many obstacles in accessing emergency healthcare. Legal uncertainties, economic constraints, language differences, and cultural disparities lead to delayed medical care and thereby exacerbate health inequities. Addressing the healthcare needs of this vulnerable group is crucial for both humanitarian and public health reasons. Comprehensive strategies are needed to ensure equitable health outcomes.

**Objective:**

This study aimed to identify and analyze the barriers undocumented immigrants face in accessing emergency healthcare services and the consequences on health outcomes.

**Methods:**

We used a scoping review methodology that adhered to established frameworks. Utilizing MEDLINE/PubMed, Embase, Web of Science, PsychoInfo, and the Cumulative Index to Nursing and Allied Health Literature (CINAHL), we identified 153 studies of which 12 focused on the specific challenges that undocumented immigrants encounter when accessing emergency healthcare services based on the inclusion and exclusion criteria.

**Results:**

The results show that undocumented immigrants encounter significant barriers to emergency healthcare, including legal, financial, linguistic, and cultural challenges. Key findings were the extensive use of emergency departments as primary care due to lack of insurance and knowledge of alternatives, challenges faced by health professionals in providing care to undocumented migrants, increased hospitalizations due to severe symptoms and lack of healthcare access among undocumented patients, and differences in emergency department utilization between irregular migrants and citizens. The findings also serve as a call for enhanced healthcare accessibility and the dismantling of existing barriers to mitigate the adverse effects on undocumented immigrants' health outcomes.

**Conclusions:**

Undocumented immigrants' barriers to emergency healthcare services are complex and multifaceted and therefore require multifaceted solutions. Policy reforms, increased healthcare provider awareness, and community-based interventions are crucial for improving access and outcomes for this vulnerable population. Further research should focus on evaluating the effectiveness of these interventions and exploring the broader implications of healthcare access disparities.

## Introduction

People who live without legal authorization in a foreign country form a significant global demographic [1]. The terms "immigrant" and "migrant" are often used interchangeably in this context; however, "immigrant" typically refers to individuals who move to another country with the intention of permanent settlement, whereas "migrant" can refer to those who move temporarily, often for work, and may not intend to stay permanently [2]. Estimates suggest there are approximately 281 million international migrants worldwide, a substantial portion of whom lack legal status in their host countries [3]. For instance, in the United States alone, it is estimated that there are around 10.5 million undocumented immigrants, representing about 3.2% of the total U.S. population [4]. Similarly, in the European Union, there are an estimated 3.9 to 4.8 million undocumented migrants [5].These individuals face many obstacles in accessing healthcare. Such obstacles include lack of health insurance, fear of deportation, ineligibility for government programs, and language and cultural differences [1, 6–14]. Addressing their healthcare needs is crucial not only from a humanitarian perspective but also for public health, as their exclusion from healthcare systems has serious consequences [15, 16].

Studies found that financial barriers to healthcare included high out-of-pocket payments, high service prices, fragmented financial support, limited funding capacity, fear of deportation, and delayed referral [12, 17]. Geographic challenges also play a role, with many migrants living in areas where healthcare facilities are either overwhelmed or scarce. These barriers hinder not only access to routine care but also emergency services, contributing to wider public health concerns [7, 12, 17–19].

In emergency care situations, undocumented immigrants face even greater challenges. They often avoid essential treatment due to financial problems and fear of legal actions [1, 6, 10, 12, 17, 18]. Even when they do seek emergency care, they often encounter language and cultural differences that can lead to misunderstandings and inappropriate treatment [7, 12]. This avoidance of essential care not only endangers their health but also affects the health of the community [10, 11, 13].

Although extensive searches were conducted, no systematic reviews were found that specifically addressed the difficulties undocumented immigrants have in accessing emergency care. The phrase "No Papers, No Treatment," used in the title of this study, reflects the harsh reality that undocumented immigrants often face when seeking healthcare. This phrase, which has been echoed in various advocacy platforms and public discussions, encapsulates the severe barriers to care that this population experiences. This scoping review aims to bridge this gap by examining those very challenges. The objectives of this review are threefold: 1) to identify the specific barriers encountered; 2) to understand the reported consequences of these barriers on undocumented immigrants; and 3) to examine the solutions that have been proposed to improve their access to emergency care. By undertaking this study, we aim to provide a foundational understanding of the complexities involved in access to emergency healthcare for undocumented immigrants, thereby contributing to the body of knowledge and suggesting pathways for future research and policy development. This is the first study to address this neglected issue in healthcare research and policy.

## Methodology

This scoping review was designed by integrating the methodologies described by Arksey and O'Malley (2005) [20] and further refined by Levac et al. (2010) [21]. The research team consisted of two reviewers, who are also the authors of this work. These reviewers formulated the main research objectives and outlined the review by defining the search terms, identifying the databases for the literature search, and establishing the inclusion and exclusion criteria. We selected the MEDLINE/PubMed, Embase, Web of Science, PsychoInfo, and Cumulative Index to Nursing and Allied Health Literature (CINAHL) databases due to their extensive coverage of medical, psychological, and health literature. The search terms were chosen to cover a wide array of relevant components ("emergency" OR "emergency care") AND ("undocumented immigrants" OR "illegal immigrants" OR "unauthorized immigrants" OR "undocumented migrants" OR "irregular migrants"). This ensured the inclusion of literature that specifically addressed barriers faced by undocumented immigrants in accessing emergency care.

**Fig. 1:**
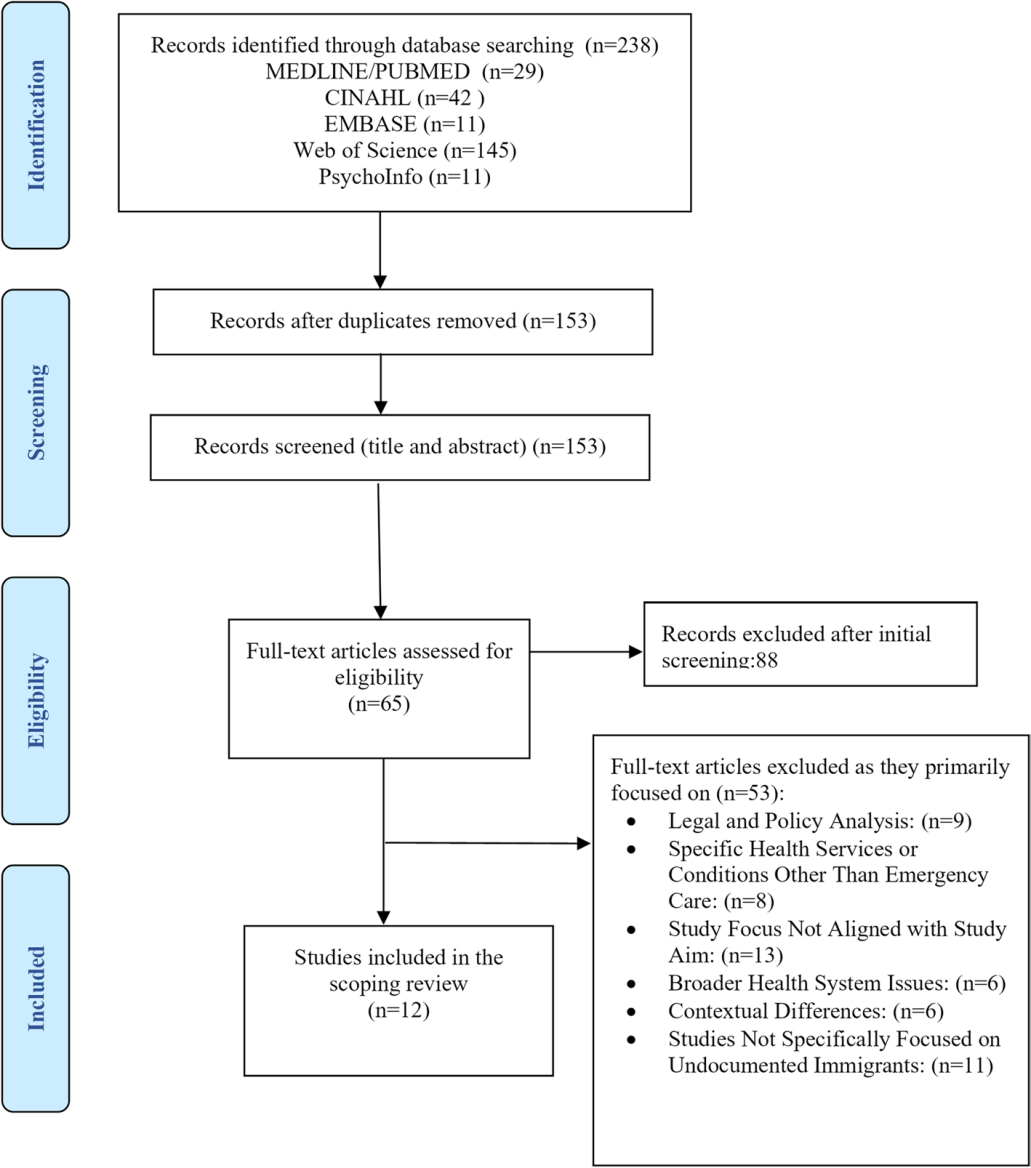
PRISMA

The search and selection processes were conducted by both reviewers. Duplicates were removed, followed by two parallel and separate screenings of titles and abstracts by each reviewer. The full-text review and data extraction were also performed independently by each reviewer, with any disagreements resolved through discussion. Our scoping review did not include a formal quality assessment of the included studies, in line with Arksey & O'Malley's (2005) [20] recommendations for scoping reviews. We limited our review to peer-reviewed research articles that examined undocumented immigrants' barriers to emergency care and were published in English up to February 29, 2024. Studies were excluded if they did not focus on undocumented immigrants in accessing emergency care, were not related to undocumented immigrants, were not based on empirical research, or were published in languages other than English. This extensive selection process resulted in a total of 12 studies for the final review (Fig. 1).

All findings were entered in EndNote (version 21). The data from the included studies, which related to characteristics such as author, publication year, study design and participants, sample size, study purpose, and key findings were extracted and charted by the first author in Excel to address the research objectives.

## Results

This review uncovered 12 studies on emergency care use by undocumented individuals in the United States [13, 18, 22–24], Switzerland [25], Denmark [9], French Guiana [10], Israel [19], Norway [15, 26], and Spain [16]. The methodologies of the studies varied. They encompassed six cross-sectional surveys [10, 13, 18, 19, 22, 24], one prospective cohort design [25], one historical cohort study [15], one case-control study [23], one observational cross-sectional study [26], and two qualitative studies [9, 16]. Notably, the study by Jiménez-Lasserrotte et al. (2023) included valuable insights from nurses who were directly involved in the care of child migrants, highlighting their critical role in health and social triage, as well as in addressing the immediate health needs of this vulnerable population. Sample sizes varied significantly across these studies, ranging from small-scale qualitative interviews with 12 participants [9] to large-scale analyses involving over half a million individuals [19]. The studies were published between 1996 and 2023.

Key findings were the excessive use of emergency departments for primary care due to lack of insurance and knowledge of alternatives [22], challenges faced by health professionals in providing care to undocumented migrants [9], increased hospitalizations due to severe symptoms, and lack of healthcare access [10, 23], and differences in emergency department utilization between irregular migrants and citizens [19] (Table 1).

**Table 1:** Summary of included studies

**Author/year**	**Study design and participants**	**Sample size**	**Study purpose**	**Findings**
Chan et al., 1996, United States [22]	Cross-sectional: Undocumented Hispanic pa-tients aged 0-60 + years presenting at an emergency department	227	Investigate the reasons undocumented persons seek care in the US	The majority had no insurance and used the emergency department as their primary source of care 51% were unaware of other sources of care, with 44% finding no acceptable alternative to the emergency department 61% cited a lack of health insurance as a reason for seeking care 36% had difficulty obtaining care elsewhere due to their status
Nandi et al., 2008, United States [13]	Cross-sectional: Undocumented Mexican im-migrants aged 18-60 years residing in New York City	431	Assess access to and use of health services among undocumented Mexican immigrants	Factors associated with health insurance coverage included fewer adults in residence, higher formal income, and linguistic acculturation Female gender, higher formal income, and health insurance coverage were associated with access to a regular health care provider Higher education and income levels were associated with emergency department care
Wolff et al., 2008, Switzerland [25]	Prospective cohort: Un-documented pregnant mi-grants aged 18-40 years vs. pregnant wom-en with legal residency	161	Compare the use of preventive measures and pregnancy care between undocumented pregnant migrants and women from the general population of Geneva	Undocumented migrants had more unintended pregnancies, less preventive care, delayed prenatal care, and higher exposure to violence during pregnancy compared to women with legal residency
Akincigil et al., 2011, United States [18]	Cross-sectional: Undocumented Mexican im-migrants aged 18 + years applying for a Matricula Consular	4836	Examine emergency room use by undocumented Mexican immigrants and their sources of health care information	38% of respondents reported using an ER for primary medical care, declining with time spent in the US ER use varied significantly by region Those receiving information from churches reported less ER use
Jensen et al., 2011, Denmark [9]	Semi-structured interviews: General practi-tioners (GPs) and emergency room (ER) physicians (No age infor-mation provid-ed	12 9 GPs and 3 ER physicians	Explore how health professionals navigate and experience providing treatment for undocumented migrants in the Danish health care system	Health professionals described challenges in providing care due to administrative barriers, financial issues, and lack of clear guidelines for treating undocumented migrants GPs faced difficulties in referring migrants for further care due to lack of a social security number and insurance
Maldonado et al., 2013, United States [24]	Cross-sectional: Undocumented Latino immi-grants (UDLI), Latino legal residents (LLR), non-Latino legal residents (NLLR) aged 18 +	1007 UDLI: 314 LLR: 373 NLLR: 320	Examine knowledge, beliefs about reporting of illegal immigrants, fear of discovery, and sources of fear in the ED	12% of UDLI expressed fear of discovery and deportation Belief that medical staff report UDLI and recent immigrants are risk factors for this fear Family, friends, and media are primary sources of these concerns
Jolivet et al., 2014, French Guiana [10]	Cross-sectional observational: Patients 16 years and older presenting to the Saint-Laurent du Maroni Hospi-tal's Emergen-cy Department (ED)	177	Describe the characteristics of undocumented patients using the ED, compare sociodemographic and medical characteristics based on residency status, and analyze factors associated with hospitalization	27.7% of ED patients were undocumented migrants They were more often hospitalized than other patients due to more severe symptoms, poorer access to health insurance, greater distance from their home to the hospital, and poorer French language skills
Madden & Qeadan, 2017, United States [23]	Case-control: Patients aged 18-90 years admitted to University of New Mexico Hospital's ER for dialysis	4104	Measure likelihood of ER dialysis among new/undocumented immigrants compared with other patients	Hispanic patients who used an interpreter (proxy for new/undocumented immigrants) were significantly more likely to be admitted for ER dialysis compared to other patients, likely due to ineligibility for Medicaid
Shachaf et al., 2020, Israel [19]	Cross-sectional: Irregular mi-grants (IM) and Israeli citizens (IC) aged 18-65 + years	549,713 IM: 21,495 IC: 528,218	Compare ED use by IM and IC, including demographic characteristics, hospitalization rates, and medical conditions	IM were younger, more often males, self-referred, attended during evenings/weekends, and mainly suffered occupational injuries They stayed longer in the ED but had lower hospitalization rates compared to IC Disparities suggest the need for improved community care access for IM
Eick et al., 2022, Norway [15]	Historical cohort: Preg-nant women aged 18–49 attending urban non-government (NG) clinics	500	Investigate utilization of antenatal health care services at NG clinics and assess maternal and perinatal outcomes	Pregnant undocumented women received substandard antenatal care and had a high risk of adverse pregnancy outcomes, despite low occurrence of comorbidities They were referred for induced abortion at a total rate of 28.4%
Eick et al., 2023, Norway [26]	Observational, cross-sectional: Pregnant un-documented migrants aged 18–49	225	Compare consultations of pregnant undocumented migrants to residents	Pregnant undocumented migrants had higher urgency levels and hospitalization rates compared to residents
Jiménez-Lasserrotte et al., 2023, Spain [16]	Qualitative study: Healthcare providers from Spanish Red Cross aged 24–60	21	Describe and understand the health and emergency care needs and processes for irregular child migrants arriving in Spain via small vessels	Healthcare providers emphasize the importance of child protection, value the family unit, and ensure children's safety Highlights were the multidisciplinary approach and the challenges in care due to diverse migrant statuses and needs

## Barriers to accessing emergency healthcare

Barriers to accessing emergency care were broadly categorized under six themes: linguistic, financial, legal, cultural, health literacy, and other (Table 2).

**Table 2:** Barriers to accessing emergency care

**Author/ year**	**Barriers**					
	**Linguistic**	**Financial**	**Legal**	**Cultural**	**Health literacy**	**Other**
Chan et al., 1996 [22]	NS	Lack of health insurance, restricted medical benefits	Fear of deportation; difficulty obtaining care due to undocumented status	NS	Unaware of alternative sources of care; lack of knowledge of where to seek help	Previous care at hospital perceived as better
Wolff et al., 2008 [25]	NS	Lack of health insurance, high costs associated with healthcare	Undocumented status contributing to delayed prenatal care	NS	Lack of knowledge about emergency contraception, under-utilization of preventive measures such as Pap test	NS
Nandi et al., 2008 [13]	NS	Lack of health insurance due to undocumented status	Fear of discovery and deportation affecting access	NS	NS	Discrimination experiences related to race, language, and immigrant status; lack of social support; working as a day laborer; sending remittances hindering ability to afford care
Akincigil et al., 2011 [18]	Limited English proficiency impacting communication	High uninsured rates due to undocumented status	Fear of deportation affecting willingness to seek care	Mistrust and potential discrimination in healthcare settings	Lack of knowledge regarding available healthcare services	Transportation issues; lack of childcare preventing access; structural healthcare system barriers
Jensen et al., 2011 [9]	Communication problems due to language differences	Lack of health insurance and financial constraints	Lack of formal entitlements to primary care, such as a health insurance card	NS	Lack of knowledge about available healthcare services	Administrative hurdles; complications related to lack of previous medical records; contact persons' and professionals' uncertainty on how to handle situations including further referrals
Maldonado et al., 2013 [24]	Poor English proficiency	Lack of health insurance	Fear of being reported to authorities	NS	Lack of knowledge about rights to care and healthcare system	Fear of not receiving medical care; reliance on friends/ family and media for information
Jolivet et al., 2014 [10]	Poor French language skills	Lack of health insurance, high costs associated with healthcare	Undocumented status contributing to delayed prenatal care	NS	Limited knowledge about available healthcare services	Geographical remoteness; lack of health-care facilities in border areas; complex health insurance systems for undocumented migrants
Madden & Qeadan, 2017 [23]	Low English language proficiency	Lack of health insurance due to ineligibility for Medicaid	Legal status preventing qualification for Medicaid	NS	NS	Absence of a uniform national policy; lack of access to routine outpatient dialysis
Shachaf et al., 2020 [19]	NS	Limited access due to exclusion from national health insurance; financial constraints due to unemployment or underemployment	Legal status restricting access to healthcare and insurance	Cultural differences NS but implied through contextual factors	Limited knowledge about healthcare services; inadequate orientation to ambulatory treatment alternatives	NS
Eick et al., 2022 [15]	NS	Excluded from general practitioner and reimbursement schemes	Legal status impacting access and care; excluded from Norwegian public healthcare schemes	NS	NS	Structural vulnerabilities; poor working and living conditions; migratory challenges; psychosocial hardship
Eick et al., 2023 [26]	NS	NS	Restricted access due to lack of Norwegian identity number, exclusion from general practitioner and reimbursement schemes	NS	Lack of knowledge about rights and where to seek help	NS
Jiménez-Lasserrotte et al., 2023 [16]	Communication difficulties	NS	Irregular status complicating access and care	Cultural misunderstandings and needs are unmet due to a lack of cultural mediators	Unfamiliarity with healthcare rights and services	Trust issues; complexity of migrant situations

*NS* Not specified

Lack of health insurance [9, 10, 13, 19, 22–25], restricted medical benefits [22], high costs associated with healthcare [10, 25], financial constraints due to unemployment or underemployment [19]; and exclusion from general practitioner and reimbursement schemes [15] were reported as the financial barriers to emergency care.

Most of the legal barriers were related to one's undocumented status and lack of entitlements, such as a health insurance card or identity number [9, 10, 15, 16, 19, 22, 23, 25, 26]. Fear of being reported to authorities [13, 22, 24] was mentioned in three studies. Administrative hurdles and systemic healthcare challenges, which include complications due to lack of proper documentation or previous medical records and the inefficiencies within the healthcare system itself, were also reported [9, 15, 26].

Transportation issues and lack of childcare were among the other barriers that prevented timely access to emergency healthcare [18]. Geographical remoteness and the complexity of health insurance systems [10], the patchwork system of safety net care (which is especially relevant to emergency renal disease care and the inconsistency in healthcare policies) [23], and structural vulnerabilities such as poor working and living conditions [15, 26], were other assorted factors affecting the migrants’ accessibility and utilization of healthcare services.

### Consequences of barriers

The costs of these identified barriers were increased reliance on emergency departments as primary care sources, higher rates of unfunded visits, and delays in treatment [22]; unintended pregnancies, delayed prenatal care, increased exposure to violence during pregnancy [25]; and limited access resulting in neglect of preventive care and excessive emergency service use [13, 18]. The researchers also identified disparities such as: unequal access to primary care, delayed treatment, and administrative burdens [9]; fears leading to delayed healthcare access and higher emergency severity [24]; extended emergency department stays and lower hospitalization rates for non-severe conditions [19]; substandard antenatal care and related risks [15, 26]; more severe conditions upon hospital arrival and higher hospitalization rates [10]; and specific issues such as increased emergent dialysis usage and associated costs [23] (Table 3).

**Table 3:** Consequences of barriers, solutions, and future research directions

**Author/year**	**Consequences of barriers**	**Suggested solutions**	**Research gaps and future directions**
Chan et al., 1996 [22]	Increased ED use as primary care; higher rates of unfunded visits; possible delayed care leading to more acute conditions requiring costlier treatment	NS	Need to understand how undocumented immigrants access healthcare, as well as the impact of policies and strategies to improve access to regular medical services for undocumented immigrants
Wolff et al., 2008 [25]	More unintended pregnancies; delayed prenatal care; higher violence exposure during pregnancy	Provide services free or at low charge; provide language and culturally appropriate education	Need to determine how illegal status influences access to care for improving prenatal care utilization among undocumented migrants
Nandi et al., 2008 [13]	Underuse of preventive care and reliance on emergency services for health needs	Policies to address factors limiting access to care; large-scale political solutions; improve social and economic resources; engage in the formal economy and navigability of the US healthcare system	Need to understand the socioeconomic determinants to healthcare access among undocumented immigrants and develop effective interventions
Akincigil et al., 2011 [18]	Overuse of emergency services; underuse of preventive care and strain on hospital systems	Enhance information dissemination through trusted community resources like churches	Investigate variations in ER use by location and the impact of information sources on healthcare-seeking behavior; explore how structural healthcare system differences and transportation affect ER use
Jensen et al., 2011 [9]	Unequal access to primary care; delayed treatment; increased administrative work	Clarify legal rights and responsibilities; improve access to primary care; provide language support	More research on access and quality of care for undocumented migrants; development of clear guidelines for healthcare professionals
Maldonado et al., 2013 [24]	Delayed healthcare access; fear influencing health care access	Inform patients about confidentiality in ED; educate the community on health rights; clarify non-reporting practices by medical staff	Learn the impact of fear on healthcare access; strategies to ensure immigrants know their rights and the healthcare system's confidentiality
Jolivet et al., 2014 [10]	More severe cases on arrival; higher hospitalization rates	Improve access to healthcare for undocumented migrants; provide free or low-cost health insurance	Need to understand the health needs and barriers faced by undocumented migrants, particularly in areas with significant migrant populations
Madden & Qeadan, 2017 [23]	Increased use of emergent dialysis; higher healthcare costs; delayed care	Create safety net chronic outpatient dialysis programs; include immigrants in Medicaid; provide maintenance dialysis to all patients regardless of status	Explore how immigration status intersects with ethnicity to affect ESRD care; assess socioeconomic characteristics influencing health care access disparities
Shachaf et al., 2020 [19]	Longer ED stays; lower hospitalization rates; non-severe medical conditions; occupational hazards	Improve access to primary care; shift staff to busy hours; improve communication; consider "social residency" for healthcare access	Need for more research on migrants' healthcare access, effective community care strategies for migrants
Eick et al., 2022 [15]	Substandard antenatal care; high risk of adverse pregnancy outcomes; high rate of induced abortions	Increase attention to structural vulnerabilities; ensure accessible and adequate antenatal care for undocumented women	Need to explore comprehensive care pathways; assess structural vulnerabilities affecting universal health coverage
Eick et al., 2023 [26]	Increased risk of high-level urgency and hospitalization; delays in seeking care leads to worse conditions	Increase access to primary and emergency care; strive towards equity in antenatal care	Understand the effect of legal status on healthcare use; strategies to increase antenatal care use among migrants
Jiménez-Lasserrotte et al., 2023 [16]	Increased vulnerability due to travel conditions; exposure to violence; lack of access to basic needs	Comprehensive care covering health, social, and emotional aspects; culturally adapted care; multidisciplinary and coordinated care among institutions	Need for improved protocols, especially for age determination and family verification; improved healthcare continuity

*NS* Not specified

### Suggested solutions

The studies advocate for systemic changes to improve healthcare accessibility and quality for undocumented immigrants. Free or low-cost services and culturally appropriate education [25], increased social and economic resources [13], information dissemination through trusted sources [18], legal clarification and language support [9], patient education about confidentiality and health rights [24], initiatives to better healthcare access for undocumented migrants and affordable insurance options [10], and inclusive Medicaid policies [23] were all recommended. Furthermore, comprehensive care that addresses health, social, and emotional aspects, with culturally adapted and coordinated approaches, were also suggested [16, 19] (Table 3).

### Research gaps and future directions

The studies identified several significant gaps and future research needs in healthcare access for undocumented immigrants. These include understanding the impacts of legislative measures [22], access to care without documentation [13, 25], improving prenatal care, variations in emergency room use, effects of information sources, and structural impacts on healthcare-seeking behaviors [18]. Other urgent areas for research are the impact of fear on healthcare access, ensuring understanding of a patient's rights and confidentiality, exploring health needs in regions with significant migrant populations, understanding intersections of immigration status with ethnicity in care disparities, and focusing on healthcare access and community care strategies for migrants [9, 19, 23]. Finally, investigating comprehensive care pathways, uncovering structural vulnerabilities that affect health coverage, and developing enhanced protocols for vulnerable migrant populations are imperative for future healthcare improvement and policy development [10, 24] (Table 3). Additionally, there is a notable lack of qualitative insight from undocumented immigrants/migrants themselves regarding their experiences and perspectives on accessing emergency healthcare. Future research should prioritize capturing these first-hand accounts to better understand the nuanced challenges faced by this population and to inform more effective and empathetic policy interventions.

## Discussion

This scoping review aimed to identify and synthesize research on the challenges faced by undocumented immigrants in accessing emergency healthcare. The objectives were to identify specific barriers to care, understand the consequences of those barriers, and explore proposed solutions to improve access. Despite differences in methodologies, participants, and regional focus, the studies highlighted the urgent need for systemic reform to improve healthcare accessibility for undocumented populations.

## Barriers to accessing emergency care

Ensuring equitable access to safe, well-organized, and high-quality emergency care services for all individuals in need can help mitigate health disparities [27]. However, several barriers were found that prevent undocumented immigrants from accessing emergency care. Most significantly, the fear of deportation led immigrants to avoid healthcare facilities [23, 24]. Asch et al. found that individuals who feared seeing a doctor lest they get reported to the immigration authorities were nearly four times more prone to delaying care for over two months, increasing the risk of disease transmission [28]. Brenner et al. noted that deportation fears forced undocumented immigrants with end-stage renal disease (ESRD) to seek emergency care only when their condition became life-threatening [29].

Cultural and linguistic barriers further complicate these challenges. Many immigrants rely on social media or friends for health information due to a lack of trust in healthcare systems [24]. Granero-Molina et al. [30] note that health providers struggle to provide care due to language barriers and cultural misunderstandings [30]. Additionally, transportation issues, childcare responsibilities, and systemic inefficiencies hinder timely access to care, particularly in emergencies [15, 18, 26].

Structural vulnerabilities also play a role, as immigrants often live and work in environments that limit their access to healthcare [15, 26]. DuBard and Massing emphasize that healthcare access for undocumented immigrants is further impeded by the complexity of health insurance systems [31]. These systemic barriers result in a system where undocumented immigrants rely on emergency departments, leading to overcrowding and increased costs [22, 23]. Hsia and Gil-González note that legal ambiguities and administrative barriers exacerbate challenges in providing consistent healthcare access to undocumented immigrants [32].

## Consequences of barriers

Barriers to emergency care have many consequences for undocumented immigrants. Relying on emergency departments for primary care leads to delays in treatment, worsening conditions, and higher hospitalization rates [10, 22]. Pregnant and undocumented women risk delayed prenatal care and exposure to violence [15, 25, 26]. Limited access to primary care results in untreated conditions becoming acute emergencies [19]. For patients with chronic conditions such as ESRD, limited access to regular hemodialysis forces them to rely on emergency departments for emergency-only hemodialysis EOHD, resulting in higher morbidity, mortality, and costs [23, 33]. Patients receiving EOHD often experience severe symptoms such as hyperkalemia and uremia before seeking emergency care [34]. Clinicians providing EOHD also report significant morale distress due to the substandard care they have to provide [33, 35]. In addition, cultural barriers during emergency triage contribute to inadequate care for undocumented immigrants, particularly those arriving by small boats in Europe [30]. Although our study did not specifically examine mental health conditions, it is well-documented that undocumented immigrants frequently experience significant mental health challenges due to the stress of living in uncertain conditions. This is particularly concerning in emergency department settings, where overcrowding and limited resources often result in inadequate mental health care for this vulnerable population.

## Proposed solutions

Addressing these challenges requires systemic improvements to healthcare access and quality for undocumented immigrants. Cervantes et al. [34] argue that enhancing access to primary and preventive care through free or low-cost services and culturally appropriate education can help reduce the reliance on emergency departments for non-emergency conditions [34]. Nandi et al. (2008) [13]emphasized the need for increased social and economic resources.

Legal clarification and policy changes that explicitly include undocumented immigrants in healthcare systems are essential. Improved access to primary care, coupled with patient education about their rights and the confidentiality of healthcare services, can alleviate fears related to immigration status [9, 24]. Affordable health insurance options and inclusive Medicaid (a joint federal and state program in the United States that provides health coverage to eligible low-income individuals and families) policies would significantly improve access to care and reduce the financial burden on safety-net programs [10, 23]. Brenner et al. (2021) [29] argue that systemic efforts to improve public health, reduce the effects of injury and illness, and secure access to emergency and basic health care for all must involve policies that prioritize care over immigration enforcement.

Programs that enhance access to primary care and consider broader inclusion policies can improve outcomes for undocumented immigrants [19]. The inclusion of diverse healthcare provider perspectives, such as those of nurses, as seen in Jiménez-Lasserrotte et al. (2023), is crucial for developing comprehensive care strategies that address the unique needs of undocumented populations. Addressing structural vulnerabilities, including working and living conditions, is essential for improving healthcare access and quality. Accessible antenatal care and comprehensive healthcare that addresses physical, social, and emotional needs are crucial for vulnerable populations [16]. Addressing legislative barriers and reducing administrative burdens, as highlighted by the challenges faced in Spain, is also essential for ensuring equitable healthcare access [32]. By focusing on these systemic changes, healthcare systems can better accommodate the needs of undocumented immigrants, ensuring they receive the necessary care without unnecessary legal and administrative obstacles. Cultural mediation can help to bridge gaps in understanding between healthcare providers and undocumented immigrants [30].

## Research gaps and future directions

Significant research gaps remain in understanding the full extent of healthcare challenges faced by undocumented immigrants. Further research is needed to understand the impact of legislative measures on healthcare access [22]. Additionally, studies should explore the influence of one's undocumented status on healthcare access and outcomes, especially in prenatal care [13, 25]. Comprehensive studies on emergency room use, information sources, and structural barriers to healthcare are needed [18].

More comprehensive studies on healthcare access and quality for undocumented immigrants are required to inform effective policies [9]. Addressing the impact of fear on healthcare access, along with strategies to ensure that immigrants understand their rights, is critical [24]. Research should focus on developing effective community care strategies to overcome healthcare barriers for migrant populations [19]. Understanding the structural vulnerabilities affecting health coverage is imperative for future care improvement and policy development [15, 26]. Further research should also explore the impact of administrative barriers and the challenges of policy implementation, as seen in Spain, to develop more effective solutions [32]. Additionally, research should prioritize examining the mental health challenges faced by undocumented immigrants, particularly in emergency settings. Given the limited resources in emergency departments, there is a critical need for targeted interventions that address these mental health needs to improve care and outcomes for this population.

## Limitations

This review has several limitations. First, a restriction to English-language publications may have excluded important studies published in other languages and limited the global representativeness of our findings. Second, the exclusion of gray literature sources, such as reports and conference abstracts, may have overlooked valuable insights, restricting the breadth and depth of our review. Third, the heterogeneous methodologies employed across included studies introduced variability and could have complicated direct comparison and synthesis of findings. These limitations emphasize the need for careful interpretation and draw attention to areas where methodological improvements are needed in future research.

## Conclusion

In conclusion, this comprehensive review found a diverse range of barriers faced by undocumented immigrants in accessing emergency healthcare services. Legal, financial, linguistic, cultural, and systemic factors collectively contribute to adverse health outcomes and strain emergency healthcare systems. Proposed solutions encompass policy initiatives such as enacting inclusive healthcare policies, together with community-based interventions like culturally tailored education and improved information dissemination. Further research is needed to understand the intersectionality of barriers, evaluate the effectiveness of proposed interventions, and assess the impact of legislative measures on healthcare access. By dismantling structural barriers, fostering cultural competency, and prioritizing the healthcare needs of undocumented immigrants, policymakers and practitioners can advance health equity agendas and foster a more inclusive healthcare landscape. Overall, addressing the diverse barriers to emergency healthcare access for undocumented immigrants is crucial for promoting health equity and improving public health outcomes. We will only achieve a truly healthy society when all its members, documented and otherwise, receive the care they need and deserve.

## Data Availability

All data generated or analyzed during this study are included in this published article.

## Data availability

No datasets were generated or analysed during the current study.
